# Replacing alfalfa hay with industrial hemp ethanol extraction byproduct and Chinese wildrye hay: Effects on lactation performance, plasma metabolites, and bacterial communities in Holstein cows

**DOI:** 10.3389/fvets.2023.1061219

**Published:** 2023-01-26

**Authors:** Yiqiang Wang, Qingyuan Yu, Xiaolin Wang, Jiamei Song, Modinat Tolani Lambo, Jianguo Huang, Ping He, Yang Li, Yonggen Zhang

**Affiliations:** ^1^College of Animal Science and Technology, Northeast Agricultural University, Harbin, China; ^2^Heilongjiang Wellhope Agri-Tech Co., Ltd., Harbin, China; ^3^Harbin Wellhope Trading Co., Ltd., Harbin, China

**Keywords:** industrial hemp ethanol extraction byproduct, lactation performance, plasma metabolites, bacterial community, dairy cow

## Abstract

This trial was designed to investigate the effects of industrial hemp ethanol extraction byproduct (IHEEB) and Chinese wildrye hay (CWH) replacement of alfalfa hay (AH) on digestibility, and lactation performance, plasma metabolites, ruminal fermentation, and bacterial communities in Holstein dairy cows. Nine healthy multiparous Holstein cows (parity = 3) with similar body weights (584 ± 12.3 kg), days in milk (108 ± 11.4), and milk yields (30 ± 1.93 kg; all mean ± standard deviation) were used in a replicated 3 × 3 Latin square design with 3 periods of 21 d. During each period, each group consumed 1 of 3 diets: (1) 0% IHEEB (0IHEEB); (2) 6.0% IHEEB and 1.7% Chinese wildrye hay (6IHEEB); (3) 10.8% IHEEB and 4.3% Chinese wildrye hay (11IHEEB). The diets in each group were isocaloric and isonitrogenous, with similar contents of concentrate and silage but different ratios of IHEEB and CWH to replace AH. The results showed that increasing the substitute did not affect the total-tract apparent nutrient digestibility. There was no difference in lactation performance of dairy cows fed the three diets, except for the cows' somatic cell count (SCC), which decreased with the increase in the amount of the substitute. Cannabidiol and tetrahydrocannabinol were not detected in milk samples of dairy cows in the different treatment groups. 6IHEEB and 11IHEEB-fed cows showed a linear decrease in total volatile fatty acids (VFA) and butyrate compared to the 0IHEEB cows. Plasma IL-1β content quadratically decreased with feeding IHEEB and CWH, and other blood parameters were unaffected. The rumen fluid's relative abundances of *Bacteroidota, Fibrobacterota*, and *Prevotellaceae* quadratically increased, while *Firmicutes* tended to decrease quadratically as the substitution increased. Feeding IHEEB and CWH linearly increased the relative abundances of *Firmicutes, Lachnospiraceae, Monoglobaceae*, and *Butyricicoccaceae* in the feces. As the substitution increased, the cost of dairy farming was reduced. In summary, substituting AH with IHEEB and CWH in diets did not affect the total-tract apparent nutrient digestibility, improved milk composition, and plasma immune indices. It changed the bacterial composition in rumen fluid and feces and improved dairy farming benefits.

## Introduction

With the consumption of milk and dairy products increasing worldwide, the number of dairy cattle has increased, and the problem of increasing the demand of roughage resources for dairy cattle has become increasingly predominant (1, 2). Alfalfa hay (AH) is a widely used roughage for dairy cows, but it is in limited quantities and expensive. As a result, a large number of agricultural processing byproducts are produced each year and are increasingly favored by dairy farms because of their high-quality fiber, abundant protein, and low prices (3–5).

Industrial hemp is defined as an annual herb of the cannabis genus in the cannabis family, whose flowers, seeds, stems, leaves, and roots contain <0.3% tetrahydrocannabinol (THC) (6). Industrial hemp can provide ruminants with a rich source of crude protein (CP), crude fiber, and minerals, but residues of active ingredients may pose a risk to consumers when they consume these animal products (7, 8). Industrial hemp ethanol extraction byproducts (IHEEB) are the residue of hemp's flower and leaf parts after ultrasound-assisted extraction of cannabidiol (CBD) in ethanol. They are generally considered to have no other value or use. After extraction, most cannabinoids in industrial hemp are removed (9). Growing legalization and demand are anticipated to increase the global production of hemp and its byproducts (10). Currently, the research on industrial hemp and its processing byproducts mainly focus on the application of seed and hemp seed cake in livestock and poultry, which is considered to have a good application prospect (11–13). However, the application of industrial hemp ethanol extraction byproducts in dairy cows has not been studied.

Some studies have shown that CBD has neuroprotective, antioxidant, analgesic, anti-inflammatory, and anti-anxiety properties and can reduce proinflammatory factors in plasma (14). In fact, CBD has very low toxicity in humans and other species and has not shown teratogenic or mutagenic activity (15, 16). Even though IHEEB can be used as feed for livestock and poultry, due to its processing mode of grinding leading to small particle size, IHEEB should be used in combination with long fiber feed for dairy cows. Chinese wildrye hay (CWH) is one of the common forage grasses with long fiber in northeast China. Recent experimental reports have shown that agricultural byproduct replacement of conventional feed in ruminants has an effect on nutrient digestibility, rumen fermentation, milk composition, and bacterial communities (5, 17). We hypothesized that the combination of IHEEB and CWH could replace AH, provide nutrients required for daily production of dairy cows, improve rumen fluid and feces microbiota composition, improve production performance, promote rumen fermentation, and exert anti-inflammatory effects in dairy cows. Therefore, this study aimed to evaluate the effect of replacing AH with IHEEB and CWH on digestibility, lactation performance, plasma metabolites, ruminal fermentation, and bacterial communities in Holstein dairy cows.

## Materials and methods

### Industrial hemp ethanol extraction byproduct

Alfalfa hay (AH) and Chinese wildrye hay (CWH) were provided by Heilongjiang Wellhope Animal Husbandry Co., Ltd. (Harbin, China). Industrial hemp ethanol extraction byproduct (IHEEB) was provided by Heilongjiang Zhongsheng Biotechnology Co., Ltd. (Daqing, China). The specific processing is as follows: First, the flower spikes and hemp leaves at the top of harvested industrial hemp plants were dried until the water content was about 10%. Then they were crushed with a grinder to pass through a 5-mm mesh sieve. Finally, the crushed substance was continuously ultrasound-assisted extracted in ethanol solution several times until almost all cannabinoids were dissolved in the extract. Then, the residue after extraction was recycled by drying to obtain the IHEEB.

### Animals, diets, and experimental design

This experiment was approved by Northeast Agricultural University and was carried out at Zhongwang Dairy Farm (Heihe, China) from December 2021 to February 2022. All animal care and handling procedures were done in accordance with the regulations of the Animal Welfare and Ethics Committee of Northeast Agricultural University (Protocol number: NEAUEC20210245). Nine healthy multiparous Holstein cows (parity = 3) with similar body conditions (BW = 584 ± 12.3 kg, DIM = 108 ± 11.4, milk yield = 30 ± 1.93 kg; mean ± SD) were assigned in a replicated 3 × 3 Latin square design with 3 periods of 21 d (initial 14 d of diet adaption and final 7 d of sample collection). During each period, groups consumed 1 of 3 treatment diets. The content of concentrate and silage in each treatment diet was similar, but the ratio of IHEEB and CWH to replace AH was different. The treatments were (1) 0% IHEEB (0IHEEB); (2) 6.0% IHEEB and 1.7% Chinese wildrye hay (6IHEEB); (3) 10.8% IHEEB and 4.3% Chinese wildrye hay (11IHEEB). According to the Cornell-Penn-Miner dairy model (18), the purpose of formulating an isocaloric isonitrogenous diet was to provide sufficient energy and protein for a cow producing 35 kg/d of milk containing 3.5% fat and 3.1% protein (18). During the experiment, each cow was housed in a separate enclosure with a concrete floor, dry manure and clean rice husk bedding, self-locking neck clamps in the feeding channel, smooth ceramic tiles on the feeding floor, and free drinking water. Cows were fed twice daily (0,530 and 1,730 h) at 105% *ad libitum* intake and milked thrice daily (0,500–0,530 h, 1,300–1,330 h, and 2,000–2,030 h). The feed was pushed up at least 10 times daily, especially after milking.

### Feeds and feces

The total mixed ration (TMR) delivered and refused were weighed and sampled daily for individual cows for 7 consecutive days (d 15–21) each period to calculate dry matter intake (DMI), and the particle size of TMR was measured by using the Penn State Particle Separator (19, 20). Fecal samples (500 g) were collected from the cow rectum every 9 h for 3 consecutive days (d 15, 16, and 17) and composited for each cow each period (21). Also, a portion of the fecal sample (~5 g) collected at d 17 was stored instantly in liquid nitrogen until the determination of bacterial communities. During these 3 days, all TMR, orts, feed ingredients, and fecal samples were collected, stored at −20°C, and mixed per cow for each period. Indigestible neutral detergent fiber (iNDF) concentrations in the diet and feces were used as internal markers to estimate the total-tract apparent digestibility of nutrients (22). The iNDF content in the feces, TMR, and orts was determined by *in situ* incubation for 288 h, as described by Huhtanen et al. (23). All feeds and fecal samples were dried at 55°C for 48 h, ground through a 1-mm screen, then placed in sealed bags and stored at 4°C until used for subsequent chemical composition analysis. They were analyzed for the contents of DM (method 930.15), CP (method 976.05), ether extract (method 920.39), and ash (method 942.05) according to AOAC International (24). Neutral detergent fiber (NDF) and acid detergent fiber (ADF) were analyzed using Ankom 220 Fiber Analyzer (Ankom Technology Corp., Macedon, NY), according to the methods of Van Soest et al. (25). Starch content was measured using the Megazyme Total Starch Assay Kit (K-TSTA; Megazyme International Ireland Ltd.). CBD and THC were analyzed using a high-performance liquid chromatograph with an ultraviolet detector (LC-20A; Shimadzu Corp., Japan) at National Market Regulation Technology Innovation Center (Industrial Hemp) (Qiqihar, China) (26). The lower limit of detection (LOD) and lower limit of quantification (LOQ) of cannabinoids was 0.03 and 0.1 mg/kg.

### Milk yield and composition analysis

The milk yield of each cow was recorded for 3 consecutive days (d18, 19, and 20) during the experimental period. Milk samples were collected from each cow while the cows were milked in a parallel milking parlor. According to the real milk yield of cows three times a day, milk samples (50 mL) were mixed with potassium dichromate and stored at 4°C until used for subsequent determination of milk components. The protein, fat, lactose, and milk urea nitrogen (MUN) concentrations and SCC of milk samples were analyzed by a 4-channel spectrophotometer (MilkoScan; Foss Electric, Hillerød, Denmark) at the Heilongjiang Academy of Agricultural Reclamation (Harbin, China). CBD and THC in milk were analyzed by liquid chromatography-mass spectrometry (Triple Quad 5500 + QTRAP Ready; AB Sciex Ltd., USA), according to the methods of Escrivá et al. (27). The LOD and LOQ of cannabinoids in milk were 1.5 ng/ml and 5 ng/ml.

### Blood collection and analyses

Blood samples were collected from the coccygeal vein before morning feeding on 2 consecutive days (d 20 and 21 of each period) into sodium heparin tubes. They were separated at 3,000 × *g* for 15 min at 4°C to obtain plasma and stored in a microtube at −20°C until analysis. A fully automatic biochemical analyzer was used to analyze the total protein, albumin, globulin, triglyceride, total cholesterol, urea nitrogen, and glucose in plasma (Mindray BS-420; Shenzhen Mindray Bio-medical Electronics Co. LTD). The non-esterified fatty acid (NEFA), β-hydroxybutyric acid (BHBA), total antioxidant capacity (T-AOC), total superoxide dismutase (T-SOD), catalase, glutathione peroxidase (GSH-Px), malondialdehyde (MDA), immune globulin A (IgA), immune globulin G (IgG), immune globulin M (IgM), soluble CD_3_ and soluble CD_4_ levels in plasma were determined by following the manufacturer's instructions for commercial colorimetric analysis kits (Beijing sinouk institute of biological technology). Plasma interleukin-1β (IL-1β), interleukin-6 (IL-6), tumor necrosis factor-α (TNF-α), prolactin, triiodothyronine, and thyroid hormone concentrations were determined using commercial bovine ELISA kits (Beijing sinouk institute of biological technology).

### Ruminal fluid collection and analyses

Rumen fluid (150–200 mL) was collected by gastric tube at 3 h after morning feeding on d 21 of each period. The first 100 mL of rumen fluid collected initially was discarded to prevent saliva contamination. After the rumen fluid was filtered with four layers of gauze, the pH of the filtrate was measured immediately with a pH meter (PHS-3C, Nanjing Nanda Analytical Instrument Application Research Institute). 10 mL filtrate was placed into centrifuge tubes, and 2 mL metaphosphoric acid solution (25%, wt/vol) was added immediately. After mixing, it was stored at −20°C until VFA concentration was analyzed by gas chromatography [GC-8A; Shimadzu Corp; (28)]. 0.2 mL sulfuric acid solution (50%, vol/vol) was added to another 10 ml filtrate, which was mixed and stored at −20°C until ammonia-N concentration was analyzed using the phenol-hypochlorite method (29). The microbial protein (MCP) in rumen fluid was separated by differential centrifugation according to the method of Cotta and Russell (30), and the concentration of MCP was determined by Coomassie brilliant blue method (31). Finally, 9 mL filtrate was divided into freezable tubes and immediately stored in liquid nitrogen for rumen bacterial community analysis.

### Bacterial communities

The analysis of rumen fluid and fecal samples was carried out at Biomarker Technologies Co. Ltd. using high-throughput sequencing. The DNA was extracted from the samples using an MN NucleoSpin 96 Soi DNA kit (Gene Company Limited) according to the kit's instructions. The DNA obtained from each sample was subjected to 2-step PCR amplification to construct a small-fragment sequencing library described by Jiang et al. (32). The PCR products obtained in the first step of amplification were used as templates for the second step of Solexa PCR amplification (Applied Biosystems Inc.). The V3-V4 region of the 16S rRNA gene was amplified using primers 338F (50-ACTCCTRCGGGAGGCAGCAG-30) and 806R (50-GGACTACCVGGGTATCTAAT-30) (33). The Solexa PCR products from the Solexa PCR amplification were purified using an OMEGA DNA purification column (Gene Company Limited). The purified products were quantified using a Quant-iT PicoGreends DNA Assay Kit (Gene Company Limited) following the kit's instructions. Then, the amplicons were sequenced at Biomarker Technologies Co., Ltd. using Illumina HiSeq 2500 sequencing platform (Illumina Inc.; Novaseq 6000; paired-end; 250 bp). The resulting raw image data files were analyzed by base calling and converted to the original sequenced reads. The original tag data (1,439,607 and 1,433,006 raw reads for the ruminal and fecal samples, respectively) were obtained using FLASH software [version 1.2.11; (34)]. The raw tags obtained by sequencing were filtered by Trimmomatic software [version 0.33; (35)], and then, Cutadapt software [version 1.9.1; (36)] was used to identify and remove primer sequences, and clean reads (1,430,677 and 1,423,646 clean tags for the ruminal and fecal samples, respectively) without primer sequences were obtained. We then use Usearch software [version 10.0; (37)] to perform double-ended sequence stitching on Clean Reads of each sample through overlap and then conduct length filtering on the data after stitching according to the length range of different regions. UCHIME software [version 8.1; (38)] was used to identify and remove chimeric sequences to obtain the final effective tags (1,111,160 and 1,126,303 effective tags for the ruminal and fecal samples). The tags were binned into operational taxonomic units (OTU) using the clustering program USEARCH [version 10.0; (39)] based on a 97% sequence similarity level. The obtained OTU was eventually used for taxonomic assignment. The representative sequences for each OTU were compared with the Silva (Release 128; www.arb-silva.de) database to obtain taxonomic classification at the phylum, class, order, family, and genus levels. The relative abundances of taxa at the phylum, family, and genus level were determined using QIIME software [version 1.9.1; (40)] to compare the bacterial community composition in the rumen and feces among treatments. Alpha diversity indices, including Chao, Shannon, Ace, and Simpson, were determined using QIIME software [version 1.9.1; (40)].

### Statistical analysis

Before analyses, all data were screened for normality using the UNIVARIATE procedure of SAS. All data from the experiment were analyzed in a 3 × 3 Latin square design using the Proc Mixed procedure of SAS (version 9.2; SAS Institute Inc., Cary, NC), according to the model *Y*_*ijkm*_ = μ + *T*_*i*_ + *P*_*j*_ + *C*_*k*_ + *S*_*m*_ + *E*_*ijkm*_, where *Y*_*ijkm*_ was the observation, μ was the overall mean, *T*_*i*_ was the fixed effect of the treatment, *P*_*j*_ was the fixed effect of the period, *C*_*k*_ was the random effect of the cows, *S*_*m*_ was the fixed effect of the square, and *E*_*ijkm*_ was the residual error. No carryover effects were detected (*P* > 0.05) for any data. Orthogonal polynomial contrasts were also used to analyze the linear and quadratic effects of increasing IHEEB and CWH on each variable. For this experiment, Duncan's multiple range tests were used. Significant differences were declared at *P* ≤ 0.05, and trends were defined at 0.05 < *P* ≤ 0.10.

## Results

### Roughage and diet characteristics

The three roughages differ greatly in nutrient composition (Table 1). IHEEB had higher Ash, CP, EE, Ca, and P levels than others, while CWH had the highest content of DM and NDF. Most of the nutrient composition values of alfalfa were between IHEEB and CWH, except ADF. In addition, IHEEB contains 0.03% CBD. The contents of CP, pdNDF, ether extract, Ca, and P were slightly increased, while the contents of NDF, ADF, NFC, and starch were slightly decreased with increased IHEEB and CWH supplemental levels (Table 2). Although the proportion of particles retained on the third sieve (1.18–8.0 mm) of the Penn State Particle Separator did not differ among treatments, with the proportion of replacements increasing, the proportion of particles retained on the first and second sieves decreased, whereas particles retained on the bottom pan were increased. As a result, the physically effective neutral detergent fiber (peNDF) of the 6IHEEB and 11IHEEB groups were smaller than that of the 0IHEEB group, especially the 11IHEEB group.

**Table 1:** Chemical composition of alfalfa hay (AH), Chinese wildrye hay (CWH), and industrial hemp ethanol extraction byproduct (IHEEB).

**Item**	**AH**	**CWH**	**IHEEB**
DM, %	91.6	92.0	88.7
Ash, % of DM	7.73	5.66	22.1
CP, % of DM	16.2	6.29	19.0
Ether extract, % of DM	3.14	2.09	5.25
NDF, % of DM	58.8	71.5	46.9
ADF, % of DM	43.3	37.1	27.3
Starch, % of DM	1.74	1.19	0.31
Ca, % of DM	1.63	0.91	3.57
P, % of DM	0.36	0.29	0.97
CBD, % of DM	-	-	0.03
THC, % of DM	-	-	< LOQ

**Table 2:** Ingredients and chemical composition (% of DM) of the 3 diets^a^.

**Item**	**0IHEEB**	**6IHEEB**	**11IHEEB**
**Ingredient**			
Alfalfa hay	15.1	7.40	0.00
Industrial hemp ethanol extraction byproduct	0.00	6.00	10.8
Chinese wildrye hay	0.00	1.70	4.30
Corn silage	33.4	33.4	33.4
Rumen-protected soybean meal	8.31	8.31	8.31
Wet stored corn	18.2	18.2	18.2
Corn grain	5.21	5.21	5.21
Soybean meal	7.77	7.77	7.77
Sprayed corn bran	2.45	2.45	2.45
Distillers dried grains with solubles	2.04	2.04	2.04
Corn germ meal	0.82	0.82	0.82
Rice bran meal	2.04	2.04	2.04
Molasses beet	0.41	0.41	0.41
Premix^b^	2.45	2.45	2.45
Fatty powder	1.30	1.30	1.30
Sodium bicarbonate	0.43	0.43	0.43
**Chemical composition**			
CP	16.7	16.9	16.9
NDF	31.9	31.5	31.3
ADF	20.1	19.5	18.9
pdNDF	22.3	22.0	23.0
NFC	39.6	38.9	38.5
Ether extract	4.01	4.19	4.33
Starch	27.2	27.1	27.0
Ca	0.86	0.91	0.96
P	0.39	0.42	0.46
NE_L_^c^ Mcal/kg of DM	1.62	1.61	1.61
Lys: Met	3.09	3.05	3.03
ME for milk^d^ kg/d	35.0	34.6	34.2
MP for milk^e^ kg/d	36.6	36.6	36.7
**TMR**			
>19.0 mm	9.14 ± 2.43	7.81 ± 1.55	8.12 ± 1.54
8.0–19.0 mm	42.41 ± 1.47	41.84 ± 1.86	40.19 ± 1.89
1.18–8.0 mm	10.95 ± 0.42	10.74 ± 0.40	10.67 ± 0.31
< 1.18 mm	37.27 ± 1.72	39.43 ± 1.21	40.78 ± 2.65
peNDF^f^	20.0	19.1	18.5
**Cannabinoid Content**			
CBD	-	0.002	0.003
THC	-	< LOQ	< LOQ

a 0IHEEB = 0% of DM industrial hemp ethanol extraction byproduct (IHEEB); 6IHEEB = 6.0% of DM industrial hemp ethanol extraction byproduct; 11IHEEB = 10.8% of DM industrial hemp ethanol extraction byproduct.

b Contained per kilogram of premix: Ca 185.3 g, P 58.1 g, Mg 52.7 g, Na 72.9 g, Cl 132.9 g, K 118.3 mg, S 23.6 g, Co 10.1 mg, Cu 494.3 mg, Fe 1,559.6 mg, I 25.4 mg, Mn 1,191.8 mg, Se 14.8 mg, Zn 2,377.8 mg, vitamin A 210,000 IU, vitamin D_3_ 70,000 IU, and vitamin E 1,750 IU.

c Calculated according to NRC (2001).

d Metabolizable energy allowable milk yield predictions from CPM Dairy (18).

e Metabolizable protein allowable milk yield predictions from CPM Dairy (18).

f peNDF >1.18 = physically effective NDF determined as NDF content of TMR multiplied by pef >1.18 (19).

### Nutrient intake and digestibility

As presented in Table 3, cows fed 6IHEEB and 11IHEEB showed linearly decreased intake of CP (*P* = 0.03) and ADF (*P* = 0.01), but there was no significant difference in total-tract apparent digestibility among the three groups.

**Table 3:** Intake and total-tract apparent digestibility of nutrients in Holstein cows fed the 3 diets.

**Item**	**Treatments** ^**1**^			**SEM**	***P*** **-value**	
	**0IHEEB**	**6IHEEB**	**11IHEEB**		**Linear**	**Quadratic**
**Intake, kg/d**						
DM	21.0	21.0	19.7	0.70	0.19	0.49
CP	3.39^a^	3.33^ab^	3.02^b^	0.12	0.03	0.39
NDF	5.98	5.71	5.57	0.24	0.25	0.83
Potentially digestible NDF	4.76	4.69	4.64	0.18	0.66	0.96
ADF	3.38^a^	3.06^ab^	2.84^b^	0.13	0.01	0.76
**Digestibility, %**						
DM	78.0	78.2	78.7	1.54	0.68	0.91
CP	81.2	80.2	79.6	1.40	0.41	0.91
NDF	56.7	57.6	60.5	2.43	0.28	0.75
Potentially digestible NDF	75.2	74.7	76.2	2.07	0.74	0.68
ADF	54.4	54.5	56.3	3.01	0.65	0.81

^a, b^Means within a row with different superscripts differ (P ≤ 0.05).

^1^0IHEEB = 0% of DM industrial hemp ethanol extraction byproduct; 6IHEEB = 6.0% of DM industrial hemp ethanol extraction byproduct; 11IHEEB = 10.8% of DM industrial hemp ethanol extraction byproduct.

### Milk production and components

The milk production, composition, and feed efficiency in the three diets are shown in Table 4. There was no difference in lactation performance of dairy cows fed the three diets, except that the somatic cell count (SCC) showed a decrease, and the 6IHEEB group was the lowest (linear, *P* = 0.01; quadratic, *P* = 0.05). CBD and THC were not detected in milk samples of the different treatments.

**Table 4:** Milk production, milk composition, and feed efficiency in Holstein cows fed the 3 diets.

**Item**	**Treatments** ^**1**^			**SEM**	***P*** **-value**	
	**0IHEEB**	**6IHEEB**	**11IHEEB**		**Linear**	**Quadratic**
**Production, kg/d**						
Milk	30.1	31.7	31.0	2.16	0.77	0.67
ECM^2^	30.7	31.5	32.2	2.31	0.63	1.00
4% FCM^3^	27.6	28.2	29.1	2.07	0.59	0.96
Milk fat	1.04	1.04	1.11	0.10	0.54	0.72
Milk protein	0.94	0.96	0.95	0.07	0.87	0.75
Lactose	1.45	1.51	1.47	0.10	0.84	0.65
**Composition**						
Fat, %	3.51	3.25	3.62	0.21	0.70	0.21
Protein, %	3.16	3.07	3.08	0.18	0.70	0.75
Lactose, %	4.82	4.79	4.77	0.15	0.70	0.98
MUN, mg/dL	14.7	15.4	14.8	0.84	0.89	0.37
Total solids, %	11.0	10.6	11.0	0.39	0.96	0.36
SCC, × 10^3^/mL	280^a^	191^b^	202^b^	27.6	0.01	0.05
**Feed efficiency**						
Milk/DMI	1.43	1.52	1.57	0.09	0.28	0.88
**Cannabinoid Content** ^4^						
CBD	-	ND	ND			
THC	-	ND	ND			

^a, b^Means within a row with different superscripts differ (P ≤ 0.05).

^1^0IHEEB = 0% of DM industrial hemp ethanol extraction byproduct; 6IHEEB = 6.0% of DM industrial hemp ethanol extraction byproduct; 11IHEEB = 10.8% of DM industrial hemp ethanol extraction byproduct.

^2^ECM = 0.3246 × milk yield + 13.86 × milk fat yield + 7.04 × milk protein yield.

^3^4% FCM = 0.4 × milk yield + 15 × fat yield.

^4^ND, not detected.

### Rumen fermentation

As shown in Table 5, there was a linear decrease in total VFA (*P* = 0.045), acetate (*P* = 0.05), and butyrate (*P* = 0.046) in cows fed 6IHEEB and 11IHEEB compared to cows fed 0IHEEB. With the addition of IHEEB, the content of Ammonia-N (*P* = 0.09) and the molar proportion of butyrate (*P* = 0.08) showed a quadratic increase trend.

**Table 5:** Ruminal fermentation and microbial protein synthesis in Holstein cows fed 3 diets.

**Item**	**Treatments** ^**1**^			**SEM**	***P*** **-value**	
	**0IHEEB**	**6IHEEB**	**11IHEEB**		**Linear**	**Quadratic**
pH	6.26	6.23	6.28	0.10	0.89	0.70
Ammonia-N, mg/dL	14.6	19.9	14.9	3.72	0.91	0.09
Total VFA, mmol/L	115.0^a^	99.1^ab^	92.1^b^	11.3	0.045	0.64
Acetate, mmol/L	66.6	55.4	51.5	8.09	0.05	0.57
Propionate, mmol/L	28.6	25.3	24.4	2.51	0.10	0.56
Isobutyrate, mmol/L	1.19	1.21	1.06	0.07	0.16	0.26
Butyrate, mmol/L	14.0^a^	13.2^ab^	11.3^b^	1.13	0.046	0.60
Isovalerate, mmol/L	2.48	2.19	2.04	0.31	0.17	0.80
Valerate, mmol/L	2.18	1.87	1.84	0.20	0.11	0.44
**Molar proportion of VFA, mol/100 mol**						
Acetate	57.1	55.7	55.7	1.93	0.29	0.50
Propionate	25.4	25.6	26.6	1.70	0.43	0.75
Isobutyrate	1.10	1.23	1.18	0.10	0.35	0.29
Butyrate	12.4	13.4	12.2	0.52	0.86	0.08
Isovalerate	2.12	2.22	2.23	0.12	0.46	0.71
Valerate	1.92	1.89	2.01	0.11	0.49	0.54
Acetate:propionate	2.30	2.22	2.15	0.21	0.35	0.96
Microbial protein, mg/ml	4.47	5.50	3.78	0.77	0.30	0.53

^a, b^Means within a row with different superscripts differ (P ≤ 0.05).

^1^0IHEEB = 0% of DM industrial hemp ethanol extraction byproduct; 6IHEEB = 6.0% of DM industrial hemp ethanol extraction byproduct; 11IHEEB = 10.8% of DM industrial hemp ethanol extraction byproduct.

### Plasma metabolites

The plasma biochemical, antioxidant, immune, and hormone indices did not differ among treatments; however, plasma IL-1β content decreased (linear, *P* = 0.05; quadratic, *P* = 0.01) with the increase in the substitute (Table 6).

**Table 6:** Plasma metabolites in Holstein cows fed the 3 diets.

**Item^1^**	**Treatments** ^**2**^			**SEM**	***P*** **-value**	
	**0IHEEB**	**6IHEEB**	**11IHEEB**		**Linear**	**Quadratic**
**Biochemical indices**						
Total protein, g/L	82.2	80.0	84.1	3.87	0.61	0.35
Albumin, g/L	32.8	32.0	32.6	1.50	0.91	0.64
Globulin, g/L	49.4	48.0	51.5	5.17	0.67	0.56
Triglyceride, mmol/L	0.18	0.18	0.20	0.01	0.35	0.84
Total cholesterol, mmol/L	4.65	4.87	4.90	0.27	0.52	0.77
Urea nitrogen, mmol/L	5.75	6.21	6.00	0.42	0.53	0.33
Glucose, mmol/L	4.03	3.98	3.95	0.07	0.38	0.89
NEFA, mmol/L	0.23	0.27	0.26	0.03	0.33	0.55
BHBA, mmol/L	0.50	0.51	0.46	0.05	0.56	0.61
**Antioxidant indices**						
T-AOC, U/mL	7.01	7.07	7.24	0.36	0.15	0.68
T-SOD, U/mL	82.1	82.9	83.0	4.63	0.78	0.91
Catalase, U/mL	48.8	49.2	49.4	3.08	0.74	0.96
GSH-Px, U/mL	357	368	356	27.3	0.93	0.24
Malondialdehyde, nmol/mL	3.57	3.79	3.37	0.33	0.60	0.35
**Immune indices**						
IgA, g/L	1.89	1.87	1.90	0.41	0.96	0.92
IgG, g/L	10.2	9.94	10.3	1.11	0.95	0.73
IgM, g/L	1.46	1.25	1.41	0.22	0.81	0.34
IL-1β, pg/ml^a, b^	27.7^a^	22.5^b^	24.5^b^	1.99	0.05	0.01
IL-6, pg/ml	147	145	148	14.3	0.70	0.47
TNF-α, pg/ml	59.5	61.8	61.7	5.14	0.21	0.43
sCD_3_, g/L	0.26	0.25	0.26	0.02	0.91	0.45
sCD_4_, g/L	0.08	0.07	0.09	0.006	0.23	0.12
**Hormone indices**						
Prolactin, uIU/ml	371	380	387	27.7	0.27	0.92
Triiodothyronine, ng/L	1.04	1.20	1.13	0.08	0.29	0.14
Thyroid hormone, ng/L	59.5	61.1	61.4	2.28	0.54	0.81

^a, b^Means within a row with different superscripts differ (P ≤ 0.05).

^1^NEFA, non-esterified fatty acid; BHBA, β-hydroxybutyric acid; T-AOC, total antioxidant capacity; T-SOD, total superoxide dismutase; GSH-Px, glutathione peroxidase; IgA, immune globulin A; IgG, immune globulin G; IgM, immune globulin M; IL-1β, interleukin-1β; IL-6, interleukin-6; TNF-α, tumor necrosis factor-α.

^2^0IHEEB = 0% of DM industrial hemp ethanol extraction byproduct; 6IHEEB = 6.0% of DM industrial hemp ethanol extraction byproduct; 11IHEEB = 10.8% of DM industrial hemp ethanol extraction byproduct.

### Bacterial communities

The treatments did not affect the ruminal fluid bacterial OTU, coverage, Shannon, Simpson, Ace, and Chao indices (*P* > 0.10), as indicated in Table 7. There was a quadratic decrease in fecal bacterial coverage (*P* = 0.03) with the increase in substitution. The relative abundances of *Bacteroidota* (*P* = 0.01), *Fibrobacterota* (*P* = 0.03), and *Prevotellaceae* (*P* = 0.01) quadratically increased, and *Firmicutes* (*P* = 0.06) tended to decrease quadratically with IHEEB and CWH supplementation in Table 8. There was no difference in the relative abundance of rumen fluid bacteria at the genus level among the different treatments (*P* > 0.10). The relative abundances of *Firmicutes* (*P* = 0.04), *Lachnospiraceae* (*P* = 0.047), *Monoglobaceae* (*P* = 0.04), *Butyricicoccaceae* (*P* = 0.04), *unclassified_Clostridia* (*P* = 0.01), *UCG_009* (*P* = 0.04) and *unclassified_Clostridia* (*P* = 0.01) increased linearly, and *unclassified_Clostridia_UCG_014* (P = 0.02) decreased linearly with the increase in the substitute (Table 9).

**Table 7:** Microbial diversity indices for the bacterial communities in the ruminal fluids and feces of Holstein cows fed the 3 diets.

**Item**	**Treatments** ^**1**^			**SEM**	***P*** **-value**	
	**0IHEEB**	**6IHEEB**	**11IHEEB**		**Linear**	**Quadratic**
**Ruminal fluid**						
OTU^2^	464.7	456.2	463.2	51.3	0.98	0.88
Coverage	1.00	1.00	0.99	0.00002	0.55	0.10
Shannon index	7.73	7.77	7.78	0.38	0.93	0.98
Simpson index	0.99	0.99	0.99	0.006	0.97	0.99
Ace index	465.3	456.5	464.0	51.4	0.98	0.88
Chao index	466.0	456.4	464.6	51.5	0.98	0.87
**Faces**						
OTU	523.0	526.7	527.8	23.6	0.82	0.95
Coverage	1.00	0.99	1.00	0.00002	0.99	0.03
Shannon index	8.20	8.28	8.29	0.14	0.39	0.67
Simpson index	0.99	0.99	0.99	0.001	0.15	0.46
Ace index	523.7	528.6	528.8	23.8	0.82	0.90
Chao index	523.8	531.2	532.4	24.9	0.70	0.87

^1^0IHEEB = 0% of DM industrial hemp ethanol extraction byproduct; 6IHEEB = 6.0% of DM industrial hemp ethanol extraction byproduct; 11IHEEB = 10.8% of DM industrial hemp ethanol extraction byproduct.

^2^OTU, operational taxonomic unit.

**Table 8:** Relative abundances (>1.0%) of the bacterial phyla, families, and genera in the ruminal fluids of Holstein cows fed the 3 diets.

**Item**	**Treatments** ^**1**^			**SEM**	***P*** **-value**	
	**0IHEEB**	**6IHEEB**	**11IHEEB**		**Linear**	**Quadratic**
**Phylum**						
*Bacteroidota*	0.47^b^	0.54^a^	0.46^b^	0.02	0.73	0.01
*Firmicutes*	0.41	0.33	0.41	0.03	0.93	0.06
*Proteobacteria*	0.07	0.07	0.08	0.03	0.85	0.93
*Spirochaetota*	0.02	0.02	0.02	0.006	0.72	0.97
*Patescibacteria*	0.01	0.01	0.02	0.003	0.76	0.93
*Fibrobacterota*	0.002^b^	0.009^a^	0.003^b^	0.003	0.96	0.03
Others	0.01	0.01	0.01	0.003	0.92	0.82
**Family**						
*Prevotellaceae*	0.34^b^	0.42^a^	0.31^b^	0.03	0.47	0.01
*Lachnospiraceae*	0.11	0.11	0.13	0.02	0.44	0.85
*Succinivibrionaceae*	0.06	0.06	0.07	0.03	0.86	0.90
*Oscillospiraceae*	0.06	0.04	0.06	0.01	0.89	0.22
*Ruminococcaceae*	0.07	0.03	0.05	0.02	0.58	0.12
*Acidaminococcaceae*	0.04	0.04	0.05	0.007	0.21	0.70
*Rikenellaceae*	0.04	0.04	0.05	0.01	0.43	0.39
*F082*	0.04	0.03	0.04	0.006	0.99	0.48
*Muribaculaceae*	0.03	0.02	0.04	0.008	0.81	0.24
*Spirochaetaceae*	0.02	0.02	0.02	0.006	0.72	0.97
*Selenomonadaceae*	0.02	0.03	0.02	0.005	0.68	0.21
*unclassified_Clostridia_UCG_014*	0.02	0.01	0.02	0.005	0.51	0.12
*uncultured_rumen_bacterium*	0.01	0.02	0.02	0.002	0.21	0.55
*Hungateiclostridiaceae*	0.01	0.01	0.01	0.003	0.94	0.76
*Christensenellaceae*	0.01	0.008	0.01	0.004	0.99	0.23
Others	0.10	0.09	0.09	0.01	0.44	0.80
Unclassified	0.004	0.005	0.006	0.003	0.47	0.89
**Genus**						
*Prevotella*	0.20	0.26	0.19	0.04	0.80	0.17
*uncultured_rumen_bacterium*	0.08	0.07	0.08	0.01	0.72	0.35
*Prevotella_7*	0.07	0.08	0.05	0.06	0.88	0.75
*Succiniclasticum*	0.04	0.04	0.05	0.007	0.22	0.70
*Rikenellaceae_RC9_gut_group*	0.04	0.04	0.05	0.01	0.45	0.40
*Ruminococcus*	0.06	0.02	0.04	0.01	0.52	0.12
*Succinivibrionaceae_UCG_001*	0.03	0.04	0.05	0.03	0.58	0.96
*NK4A214_group*	0.04	0.02	0.04	0.009	0.76	0.14
*unclassified_Prevotellaceae*	0.03	0.04	0.03	0.005	0.88	0.30
*unclassified_Lachnospiraceae*	0.03	0.03	0.03	0.006	0.82	0.65
*Treponema*	0.02	0.02	0.02	0.006	0.72	0.97
*unclassified_Clostridia_UCG_014*	0.02	0.01	0.02	0.005	0.51	0.12
*Succinivibrionaceae_UCG_002*	0.03	0.01	0.02	0.01	0.42	0.48
*unclassified_F082*	0.02	0.02	0.02	0.005	0.41	0.48
*Prevotellaceae_UCG_001*	0.02	0.02	0.02	0.003	0.60	0.42
*Butyrivibrio*	0.01	0.02	0.02	0.008	0.40	0.94
*Lachnospiraceae_NK3A20_group*	0.01	0.01	0.01	0.004	0.76	0.84
*Saccharofermentans*	0.01	0.01	0.01	0.003	0.90	0.82
*Prevotellaceae_UCG_003*	0.01	0.01	0.01	0.003	0.95	0.84
*UCG_005*	0.01	0.009	0.01	0.002	0.86	0.53
*Selenomonas*	0.01	0.01	0.008	0.003	0.66	0.22
Others	0.20	0.19	0.20	0.01	0.86	0.71
Unclassified	0.004	0.006	0.006	0.003	0.48	0.83

^a, b^Means within a row with different superscripts differ (P ≤ 0.05).

^1^0IHEEB = 0% of DM industrial hemp ethanol extraction byproduct; 6IHEEB = 6.0% of DM industrial hemp ethanol extraction byproduct; 11IHEEB = 10.8% of DM industrial hemp ethanol extraction byproduct.

**Table 9:** Relative abundances (>1.0%) of the bacterial phyla, families, and genera in the feces of Holstein cows fed the 3 diets.

**Item**	**Treatments** ^**1**^			**SEM**	***P*** **-value**	
	**0IHEEB**	**6IHEEB**	**11IHEEB**		**Linear**	**Quadratic**
**Phylum**						
*Firmicutes*	0.64^b^	0.64^b^	0.69^a^	0.04	0.04	0.27
*Bacteroidota*	0.29	0.32	0.28	0.05	0.54	0.18
*Actinobacteriota*	0.03	0.01	0.007	0.01	0.14	0.85
*Spirochaetota*	0.02	0.006	0.007	0.006	0.19	0.36
Others	0.02	0.02	0.02	0.006	0.63	0.78
**Family**						
*Oscillospiraceae*	0.18	0.17	0.19	0.008	0.57	0.17
*Rikenellaceae*	0.11	0.13	0.11	0.02	0.98	0.24
*Lachnospiraceae*	0.08^b^	0.09^ab^	0.10^a^	0.007	0.05	0.73
*[Eubacterium]_coprostanoligenesgroup*	0.07	0.07	0.08	0.01	0.16	0.45
*Prevotellaceae*	0.07	0.07	0.06	0.02	0.33	0.58
*Monoglobaceae*	0.04^b^	0.05^ab^	0.06^a^	0.006	0.04	0.47
*Christensenellaceae*	0.05	0.06	0.05	0.02	0.95	0.24
*UCG_010*	0.05	0.05	0.05	0.008	0.93	0.90
*Muribaculaceae*	0.03	0.04	0.04	0.005	0.36	0.55
*Ruminococcaceae*	0.03	0.03	0.03	0.003	0.86	0.15
*Bacteroidaceae*	0.03	0.03	0.03	0.01	0.84	0.95
*Anaerovoracaceae*	0.02	0.03	0.02	0.006	0.78	0.17
*unclassified_Clostridia_UCG_014*	0.03^a^	0.01^b^	0.01^b^	0.009	0.02	0.18
*Bifidobacteriaceae*	0.03	0.01	0.006	0.01	0.14	0.86
*p_2534_18B5_gut_group*	0.01	0.02	0.01	0.004	0.72	0.29
*uncultured_rumen_bacterium*	0.01	0.01	0.01	0.004	0.25	0.38
*Butyricicoccaceae*	0.009^b^	0.01^ab^	0.01^a^	0.002	0.04	0.83
*Spirochaetaceae*	0.02	0.006	0.007	0.006	0.19	0.36
*unclassified_Clostridia*	0.009^b^	0.009^b^	0.01^a^	0.001	0.01	0.37
Others	0.12	0.11	0.10	0.01	0.25	0.69
Unclassified	0.006	0.008	0.01	0.004	0.11	0.79
**Genus**						
*UCG_005*	0.15	0.13	0.15	0.009	0.77	0.14
*Rikenellaceae_RC9_gut_group*	0.07	0.08	0.08	0.01	0.72	0.32
*unclassified_[Eubacterium]_ coprostanoligenes_group*	0.07	0.07	0.08	0.009	0.17	0.47
*unclassified_Lachnospiraceae*	0.06	0.06	0.08	0.007	0.05	0.25
*Monoglobus*	0.05	0.05	0.06	0.006	0.34	0.88
*Christensenellaceae_R_7_group*	0.05	0.06	0.05	0.02	0.95	0.24
*unclassified_UCG_010*	0.04	0.04	0.04	0.008	0.88	0.87
*Alistipes*	0.04	0.04	0.03	0.009	0.52	0.40
*unclassified_Muribaculaceae*	0.03	0.04	0.04	0.005	0.32	0.65
*Prevotellaceae_UCG_003*	0.03	0.04	0.03	0.01	0.27	0.31
*Bacteroides*	0.03	0.03	0.03	0.01	0.84	0.95
*unclassified_Clostridia_UCG_014*	0.03^a^	0.01^b^	0.01^b^	0.009	0.02	0.18
*unclassified_Oscillospiraceae*	0.02	0.02	0.02	0.003	0.56	0.27
*Bifidobacterium*	0.03	0.01	0.006	0.01	0.14	0.86
*unclassified_p_2534_18B5_gut_group*	0.01	0.02	0.01	0.004	0.72	0.29
*unclassified_Ruminococcaceae*	0.02	0.01	0.01	0.002	0.81	0.27
*uncultured_rumen_bacterium*	0.01	0.01	0.01	0.004	0.38	0.32
*Prevotellaceae_UCG_004*	0.01	0.01	0.01	0.003	0.92	0.54
*Family_XIII_AD3011_group*	0.01	0.01	0.01	0.002	0.90	0.12
*UCG_009*	0.009^b^	0.01^ab^	0.01^a^	0.002	0.04	0.79
*unclassified_Clostridia*	0.009^b^	0.01^b^	0.01^a^	0.001	0.01	0.37
*Treponema*	0.02	0.006	0.007	0.006	0.19	0.36
Others	0.21	0.21	0.20	0.01	0.39	0.78
Unclassified	0.006	0.008	0.01	0.004	0.13	0.84

^a, b^Means within a row with different superscripts differ (P ≤ 0.05).

^1^0IHEEB = 0% of DM industrial hemp ethanol extraction byproduct; 6IHEEB = 6.0% of DM industrial hemp ethanol extraction byproduct; 11IHEEB = 10.8% of DM industrial hemp ethanol extraction byproduct.

### Economic benefits

With the increase of IHEEB and CWH substitution, the cost of diet was reduced, resulting in lower feed cost per kilogram of milk, which greatly increased the benefit of each cow, especially 11IHEEB, which increased by $1.65 per day compared with 0IHEEB (Table 10).

**Table 10:** Economic benefits in Holstein cows fed the 3 diets^a^.

**Item**	**0IHEEB**	**6IHEEB**	**11IHEEB**
DMI, kg/d	21.0	21.0	19.7
Diet cost^b^ $/d/cow	9.48	9.18	8.36
Milk yield, kg/d	30.1	31.7	31.0
Feed cost per kilogram of milk^c^ $/kg	0.31	0.29	0.27
Milk price, $/kg	0.60	0.60	0.60
Production value^d^ $/d	18.0	18.9	18.5
Benefit^e^ $/d	8.51	9.76	10.2

^a^0IHEEB = 0% of DM industrial hemp ethanol extraction byproduct (IHEEB); 6IHEEB = 6.0% of DM industrial hemp ethanol extraction byproduct; 11IHEEB = 10.8% of DM industrial hemp ethanol extraction byproduct.

^b^Diet cost: Alfalfa hay = 351.1 $/t, IHEEB = 191.2 $/t, Chinese wildrye hay = 171.8 $/t, Corn silage = 76.2 $/t, Rumen-protected soybean meal = 793.3 $/t, Wet stored corn =134.5 $/t, Corn grain =367.5 $/t, Concentrate = 672.3 $/t, Fat peak=1792.8 $/t, Sodium bicarbonate = 464.6 $/t.

^c^Feed cost per kilogram of milk= diet cost/milk yield.

^d^Production value = milk yield × milk price.

^e^Benefit (Does not include other costs other than the cost of feed, and assuming other costs are consistent between groups) = production value–diet cost.

## Discussion

### Diet characteristics

As an emerging and highly sought-after plant in recent years, industrial hemp produces a large number of processing byproducts yearly (10). IHEEB's development as ruminant animal feed can effectively alleviate the challenge of insufficient roughage resources for dairy cattle while making the rest worth of industrial hemp better utilized. By comparing the chemical composition of the three roughage groups, it is not difficult to find that the nutrient composition of AH, such as CP and NDF, is between IHEEB and CWH, so IHEEB and CWH can replace AH in the diet by combination. In this experiment, the content of CP in alfalfa hay was lower than that reported by Ghelichkhan et al. (41), while the content of NDF and ADF was higher, which may be caused by the late variety and harvesting period. Due to extraction and processing, IHEEB contains only a very small amount of cannabinoids, much less than the safe amount (42–44). Many factors affect peNDF in feed. The most important of these is the particle size of the feed, which promotes chewing activity (45). Therefore, in this study, CWH, a long fiber feed, was used to increase the peNDF of the diet to stimulate ruminant activity and prevent adverse effects on performance, digestibility, or rumen fermentation. The peNDF of the diets in this study all met the optimal range of Llonch et al. (46). Therefore, IHEEB can be considered an unconventional roughage resource with high safety and nutritional value, and it is feasible to substitute a part of AH with CWH.

### Nutrient intake and digestibility, lactation performance and rumen fermentation

Dry matter intake did not differ significantly, and mixing with silage masked the small amount of plant aroma of IHEEB, successfully eliminating the concerns of early scholars about low palatability and showing that cows can quickly adapt and accept the new unconventional feed (13). Although there were individual differences in dietary intake among the three groups, there did not seem to be much effect on the digestive properties of the diet, indicating that according to the total-tract apparent digestibility, IHEEB can be well-digested and absorbed by the intestinal tract of dairy cows.

In this study, IHEEB and CWH substitution did not affect the milk yield or milk composition, except for the SCC, which was decreased in milk. There were no changes in milk yield and composition for the three treated cow groups, consistent with the same feed intake observed. The SCC usually refers to the total number of cells in each milliliter of milk, mostly white blood cells (macrophages, neutrophils, and lymphocytes), with a small percentage being epithelial cells shed by mammary tissue. It is an important indicator commonly used to reflect the health of the udder of dairy cows (47). In this study, the decrease in SCC may be attributed to its strong correlation with IL-1β in the blood (48). Interestingly, we found no THC or CBD in the milk; therefore, the milk can be circulated and commercialized.

In this study, rumen pH did not change with increasing IHEEB and CWH replacement which was similar to the results of Jiang et al. (32). Rumen fluid ammonia-N concentration is related to the degradation of dietary protein in the rumen, which is utilized by rumen microorganisms. Rumen ammonia-N levels indicate more unutilized ammonia in the rumen of cows receiving the 6IHEEB diet than those receiving the other diets (49). In this study, the decrease in total VFA may be attributed to the small TMR particle size with the substitution of IHEEB and CWH in the diet, and the potential NDF could have escaped rumen fermentation and entered the post-intestinal digestion, leading to the decrease in rumen VFA production (50). In addition, different diet compositions and ruminal degradation characteristics may also be responsible for the lower VFA in dairy cows after ingestion of alternative diets. It has been suggested that IHEEB contains lower ruminal effective degradation rates compared to AH (51). Acetate and butyrate are precursors of milk fat synthesis (52). In this experiment, their concentration decreased in rumen fluid with the increase in replacement amount, but the milk fat content was not affected, possibly because dietary carbohydrates can also be fermented into VFA in the posterior gut for milk composition synthesis (53). We also found that with the substitution of IHEEB and CWH in the diet, the molar ratio of butyrate showed a curve-increasing trend, which may be caused by the change in the abundance of bacteria producing butyrate in rumen fluid, such as *Prevotellaceae* (54).

### Plasma metabolites

In this experiment, the three diets had little effect on the plasma parameters of dairy cows, except that with the increase of IHEEB and CWH substitution, IL-1β content showed a decrease, which could be associated with the slight CBD content of IHEEB. The CBD is the main non-psychoactive component of cannabinoids extracted from the industrial hemp plant (14). Numerous studies have shown that CBD treatment can reduce proinflammatory cytokines, reduce IL-1β levels in the blood, and alleviate disease or disability (55, 56). Studies have shown that CBD decreases the transmigration of blood leukocytes by downregulating the expression of IL-1β (57). The IL-1β activates a large number of neutrophils and monocytes into the mammary gland, at which time the number of somatic cells is greatly increased, resulting in mammary gland redness and inflammation caused by an immune response, so that mammary gland cells cannot work normally and have an inhibitory effect on lactation in dairy cattle (58). In addition, we did not observe any obvious effects of CBD on antioxidant indexes, and IHEEB and CWH replacement of AH did not change the plasma hormone and biochemical parameters of dairy cows.

### Bacterial communities

In this experiment, there was no significant difference in species diversity index between rumen fluids and feces, indicating that the three treatments did not affect the overall microbial species number, species abundance, and species evenness. The changes we observed in the abundance of bacterial communities were related to the composition of the diet. As mentioned above, the change in the ratio of the three roughages, especially the smaller IHEEB particles, resulted in more digestion passing through the rumen, which resulted in a change in the bacterial community in rumen fluid and feces (50).

The clustering results showed that 14 phyla, 21 classes, 38 orders, 59 families, 135 genera, and 166 species belonging to bacteria were identified in the ruminal fluid samples. By comparing the microbial community composition of the rumen at the phylum level, it can be found that *Bacteroidota, Firmicutes*, and *Proteobacteria* occupy the absolute dominant position, which is consistent with the research results of other scholars (59). The only difference is that in this experiment, *Bacteroidota* accounted for a larger proportion and showed a quadratic increase, while *Firmicutes* showed a quadratic decrease trend, and there was a negative correlation between the abundance of *Bacteroidota* and *Firmicutes* (60). Usually, healthy cows had more *Bacteroidota*, fewer *Firmicutes*, and more *Prevotellaceae* in their rumen fluid than cows with mastitis (61). The enhancement of *Prevotellaceae* may increase the abundance of short-chain fatty acids, especially butyrate, which can act as an energy substrate for intestinal epithelial cells (54). This may explain the quadratic increase in the molar ratio of butyrate observed in this experiment. Our results showed no change in the abundance of *Proteobacteria*, mainly composed of Gram-negative bacteria, and played a role in rumen nitrogen metabolism, indicating that IHEEB and CWH supplementation did not affect substrate protein processing ability (62). Therefore, the MCP content was similar among the three groups in this experiment. *Fibrobacterota* is the main cellulose-decomposing bacteria in the rumen of dairy cows. The increase of substitution enhanced the abundance of *Fibrobacterota* and increased the ability of cellulose decomposing in the rumen fluid in this experiment (63). Furthermore, starch, protein, peptides, hemicellulose, and pectin can be metabolized by rumen *Prevotellaceae* (64). The increased relative abundance of *Prevotellaceae* in the rumen fluid is beneficial for improving the degradation rate of diets, especially the concentrate part (65). This is consistent with the results of this experimental study, in which the 6IHEEB group had a higher VFA than the other group of the replacement diet.

The clustering results showed that a total of 11 phyla, 17 classes, 35 orders, 66 families, 149 genera, and 157 species belonging to bacteria were identified in the fecal samples. As previously mentioned, with the increase of IHEEB and CWH substitution, more dietary nutrients could enter the posterior gut for digestion and thus have certain effects on intestinal microbes. In this study, with the increase of substitution, the abundance of *Firmicutes* in feces increased, and it was always the phylum with the largest abundance, which was consistent with the study of Liu et al. (66). At the family level, the abundance of *Lachnospiraceae, Monoglobaceae, Butyricicoccaceae*, and *unclassified_Clostridia* increased, and the abundance of *unclassified_Clostridia_UCG_014* decreased as IHEEB and CWH were added. They all belong to the *Firmicutes*, and their increase is the main reason for the increase in *Firmicutes* (67). The increased *Lachnospiraceae* content may mean that more nutrients can be absorbed by producing more volatile fatty acids (68). Kim et al. (69) found that *Monoglobaceae Monoglobus* had a strong pectin degradation ability, which could decompose more pectin in the diet. *Clostridia* is associated with glycan degradation potential, and its increase can help animals avoid glycan loss in this study (70). All these results suggest that by increasing the substitution level, more nutrients can be efficiently digested and absorbed in the large intestines, which may explain the lack of difference in the total-tract apparent digestibility and milk components among the three groups of cows.

### Economic benefits

As an important part of dairy farm income, milk yield, milk composition, and feed cost are critical. The profitability of dairy farms can be improved by increasing milk production and improving milk composition to obtain a better price and reduce the cost of farming, such as feed costs (71). This study showed that replacing AH in the diet with a lower-priced combination of IHEEB and CWH induced no change in milk composition, resulting in the same price of milk produced and no effect on milk volume, consequently giving rise to almost the same production benefit from milk sales. Sales revenue is constant, and feed costs decrease as replacement rates increase, ultimately reducing the cost of feeding dairy farms: the less investment and constant income, the more profitable the dairy farm. In this experiment, the daily income of each cow in the 6IHEEB and 11IHEEB groups was $1.25 and 1.65 higher than that in the 0IHEEN group, making more money for the dairy farm.

## Conclusions

This study shows that IHEEB is a safe and valuable unconventional feed for dairy cows. Using IHEEB and CWH to replace 50% AH in a dairy diet can improve rumen fermentation, reduce plasma IL-1β content, reduce SCC in milk, improve rumen bacterial community and reduce feeding costs. Using IHEEB and CWH to replace 100% AH in a dairy diet can not affect the total-tract apparent nutrient digestibility, whereas it can improve the fecal bacterial community and maximize the benefit of dairy farms. In conclusion, this study not only solves the limitations of the IHEEB application but also provides data reference for a new unconventional feed for dairy cows and improves dairy farming benefits.

## Data availability statement

The datasets presented in this study can be found in online repositories. The name of the repository and accession number can be found at: NCBI; PRJNA887401.

## Ethics statement

The animal study was reviewed and approved by Animal Welfare and Ethics Committee of Northeast Agricultural University. Written informed consent was obtained from the owners for the participation of their animals in this study.

## Author contributions

YW, YL, JH, and YZ participated in the design of this study. YW, QY, XW, JS, and PH performed most of the experiments. ML revised the final version of the manuscript. YL and YZ supervised the work. All authors contributed to the article and approved the submitted version.
